# Inpp5d haplodeficiency alleviates tau pathology in the PS19 mouse model of Tauopathy

**DOI:** 10.1002/alz.14078

**Published:** 2024-06-26

**Authors:** Disha M. Soni, Peter Bor‐Chian Lin, Audrey Lee‐Gosselin, Christopher D. Lloyd, Emily Mason, Cynthia M. Ingraham, Abigail Perkins, Miguel Moutinho, Bruce T. Lamb, Shaoyou Chu, Adrian L. Oblak

**Affiliations:** ^1^ Stark Neurosciences Research Institute Indiana University School of Medicine Indianapolis Indiana USA; ^2^ Division of Clinical Pharmacology Indiana University School of Medicine Indianapolis Indiana USA; ^3^ Department of Anatomy Cell Biology & Physiology Indiana University School of Medicine Indianapolis Indiana USA; ^4^ Department of Medical and Molecular Genetics Indiana University School of Medicine Indianapolis Indiana USA; ^5^ Department of Radiology and Imaging Sciences Indiana University School of Medicine Indianapolis Indiana USA

**Keywords:** Alzheimer's disease, Inpp5d haplodeficiency, *Inpp5d*, microglia, PS19, tau pathology

## Abstract

**INTRODUCTION:**

A noncoding variant (rs35349669) within *INPP5D*, a lipid and protein phosphatase restricted to microglia in the brain, is linked to increased susceptibility to Alzheimer's disease (AD). While *Inpp5d* is well‐studied in amyloid pathology, its role in tau pathology remains unclear.

**METHODS:**

PS19 Tauopathy mice were crossed with Inpp5d‐haplodeficient (*Inpp5d+/−*) mice to examine the impact of *Inpp5d* in tau pathology.

**RESULTS:**

Increased INPP5D expression correlated positively with phospho‐Tau AT8 in PS19 mice. *Inpp5d* haplodeficiency mitigated hyperphosphorylated tau levels (AT8, AT180, AT100, and PHF1) and motor deficits in PS19 mice. Transcriptomic analysis revealed an up‐regulation of genes associated with immune response and cell migration.

**DISCUSSION:**

Our findings define an association between INPP5D expression and tau pathology in PS19 mice. Alleviation in hyperphosphorylated tau, motor deficits, and transcriptomics changes in haplodeficient‐*Inpp5d* PS19 mice indicate that modulation in INPP5D expression may provide therapeutic potential for mitigating tau pathology and improving motor deficits.

**Highlights:**

- The impact of Inpp5d in the context of tau pathology was studied in the PS19 mouse model.
- INPP5D expression is associated with tau pathology.
- Reduced Inpp5d expression in PS19 mice improved motor functions and decreased total and phospho‐Tau levels.
- Inpp5d haplodeficiency in PS19 mice modulates gene expression patterns linked to immune response and cell migration.
- These data suggest that inhibition of Inpp5d may be a therapeutic approach in tauopathies.

## 1 INTRODUCTION

Alzheimer's disease (AD) is the most prevalent type of dementia. The neuropathologic characteristics of AD are the deposition of extracellular amyloid beta (Aβ), intraneuronal neurofibrillary tangles comprised of the hyperphosphorylated protein tau, and neuronal deterioration, which collectively leads to cognitive decline and memory impairment.^1^ A wide range of genome‐wide association studies (GWAS) have provided substantial evidence that numerous genetic loci linked to increased risk of AD are associated with the immune response, which strongly implies that microglia, immune cells residing in the brain, play an essential role in the regulation of AD pathogenesis.^2^, ^3^, ^4^ Among these risk genes, the common variant within *INPP5D* has been linked to increased risk of AD (rs35349669; OR = 1.08, 95% CI = 1.06–1.11).^2^, ^5^, ^6^ *INPP5D* encodes the src homology 2 domain (SH2) containing inositol 5′ polyphosphatase 1 (SHIP1) and serves as a lipid phosphatase.^7^ SHIP1 dephosphorylates phosphatidylinositol‐3,4,5‐triphosphate, [PI (3,4,5) P3] to phosphatidylinositol‐3,4‐bisphosphate [PI (3,4) P2].^7^, ^8^, ^9^ While the expression of *INPP5D* is restricted to microglia in the brain,^8^, ^10^ it is typically found in hematopoietic‐derived cells outside the central nervous system.^7^ INPP5D acts as a crucial regulator of immune cell signaling, which limits cell functions such as proliferation, survival, phagocytosis, and cytokine production.^7^, ^8^, ^9^, ^10^, ^11^ *INPP5D* has been implicated in modulating downstream signaling pathways associated with multiple immune receptors involved in AD pathogenesis, such as TREM2, TYROBP (DAP12), CLEC7A, and FCγRs.^9^, ^10^, ^11^, ^12^

While the role of *INPP5D* has been extensively studied in peripheral tissues, its involvement in the central nervous system remains the subject of ongoing research. However, recent studies have started to elucidate the role of *INPP5D* in Alzheimer's disease. While studies have reported that elevated expression of *INPP5D* within plaque‐associated microglia correlates positively with amyloid burden,^10^ the impact of *Inpp5d* deficiency in preclinical models of Alzheimer's disease varies depending on the mouse models and timing of the inhibition. Notably, microglia‐specific deletion of *Inpp5d* in APPPS1 mice at 3 months of age increased plaque burden and recruitment of microglia toward the plaques at 6 months.^13^ Conversely, in Tyrobp‐deficient mice, characterized by *Trem2* loss of function, the haplodeficiency of *Inpp5d* appeared to restore microglia functions and engagement of microglia with amyloid plaques.^14^ Similarly, a study in 5XFAD mice that initiated the microglia‐specific deletion of *Inpp5d* at 3 weeks of age for a 2‐week period demonstrated enhanced microglia engagement with plaques and Aβ engulfment at 5 months of age.^12^ Furthermore, the haplodeficiency of *Inpp5d in* 5XFAD mice exhibited a mitigating effect on amyloid pathology, increased plaque‐associated microglia, and decreased cognitive decline at 6 to 7.5 months of age, respectively.^6^ In addition, pharmacological inhibition of *Inpp5d* in primary microglia has been shown to enhance lysosomal compartmental size and engulfment of Aβ peptide and dead neurons.^8^ Together, these studies highlight the potential of INPP5D modulation in promoting microglia functions that can be protective in response to amyloid pathology. However, its effectiveness may also be dependent on the timing during disease progression. Although significant research has been directed toward understanding the role of *INPP5D* in Alzheimer's disease, numerous studies have primarily focused on its involvement in amyloid pathology. Implications of *INPP5D* in the context of tau pathology remain unclear. Therefore, we aimed to investigate the role of *INPP5D* in tau pathology in the PS19 mouse model of tauopathy.

To study whether *Inpp5d* haplodeficiency modulates tau pathogenesis, PS19 mice were crossed with *Inpp5d*‐haplodeficient (*Inpp5d^+/−^*) mice and aged to 9 months. We observed a correlation between *Inpp5d* expression and tau pathology. Surprisingly, reducing levels of *Inpp5d* in PS19 mice mitigated tau pathology and improved motor functions. The behavioral and pathological changes were accompanied by alterations in gene expression linked to immune response and cell migration. Collectively, our data provide insights into the complex interplay between *Inpp5d* expression, tau pathology, and behavioral outcomes, suggesting that *Inpp5d* could be a potential target to mitigate tau pathogenesis and improve motor functions.

## 2 METHODS

### 2.1 Animals

All Animal experiments in this study received approval from the Institutional Animal Care and Use Committee at Indiana University. The mice were housed under a controlled 12/12‐hour light/dark cycle with access to food (Purina Lab Diet, 5K52) and water ad libitum. The study used C57BL/6J (B6; The Jackson Laboratory, 000664), and B6.129S6(C)‐*Inpp5d* ^tm1.1Wgk^/J (*Inpp5d* ^KO/WT^; mice with 50% reduction in *Inpp5d* expression, The Jackson Laboratory, 028269). The PS19 mice were back‐crossed to C57BL/6J mice for > 10 generations, resulting in the establishment of PS19 mice with C57BL/6J background in Dr. Cristian A. Lasagna‐Reeves' laboratory. This model has been carefully characterized.^15^

All experimental groups (*Inpp5d^+/+^, Inpp5d^+/−^*, PS19:*Inpp5d^+/+^*, PS19:*Inpp5d^+/−^*) included both male and female mice. Mice were anesthetized using 1.2% 2,2,2‐tribromoethanol (Avertin) and perfused with ice‐cold phosphatase buffered saline (PBS). Later, brain tissues were extracted and divided into two hemispheres. The right hemisphere was fixed in 4% paraformaldehyde, transferred to 30% sucrose for dehydration, and later used for immunohistochemistry. The left hemisphere was further dissected into the hippocampus and cortex and stored at −80^0^ freezer for biochemical studies.

### 2.2 Open field test

The open‐field test was performed using the procedure previously described in Patel H. et al. (2022).^15^ Briefly, mice were placed at the center of the dimly lit open field arena (45 × 45 × 45 cm) and allowed to explore the field for 1 h. The movements of the mice were recorded and analyzed by an automatic monitoring system (ANY‐Maze). The arena was digitally divided into distinct zones that included the central area (with sides measuring 28 cm), four wall corridors (each measuring 7 cm along the walls), and four corner squares (each with sides measuring 7 cm). The system automatically measured the time spent and total distance traveled by mice within these zones.

### 2.3 Grip strength test

The grip strength was measured using the procedure described by Patel H. et al. (2022),^15^ using the grip strength meter (Bioseb, Bio‐GS3). Following the manufacturer's instructions, mice were gently held by the tail and brought toward the device, allowing them to grasp the metal grid from two or all four paws. Afterward, mice were carefully pulled horizontally until they lost their grip, and the force applied to the grid was recorded as the highest tension and then expressed into gram units by the instrument. Two consecutive measurements were taken for the front two paws, and all four paws, and the mean value of these measurements was used for the analysis. Approximately 5 min of rest was provided to the mice between the testing sessions.

**RESEARCH IN CONTEXT**

1. **Systematic review**: The authors conducted a literature review using PubMed. The existing peer‐reviewed publications suggest that the intronic variant in the *INPP5D* gene (rs35348669) is linked to an increased risk of Alzheimer's disease. *INPP5D* is implicated in amyloid pathology in human and mouse studies. While the function of *INPP5D* is well studied in amyloid pathology, its role in tau pathology remains less explored.
2. **Interpretation**: Our results demonstrate a positive correlation between INPP5D expression and tau pathology in the PS19 mouse model of tauopathy. Mitigating tau pathology, motor impairment, and elevation in immune‐response and cell‐migration‐related genes in *Inpp5d* haplodeficient PS19 mice indicate a potential association of *Inpp5d* expression and modulation of tau pathology.
3. **Future directions**: Our findings suggest the complex interplay between the expression of *INPP5D*, tau pathology, and behavior outcomes. Further research is necessary to understand the mechanism by which *INPP5D* impacts tau pathology.

### 2.4 Immunofluorescence

Following perfusion and brain dissection, the right hemibrains of the mice were fixed overnight at 4°C using a 4% paraformaldehyde solution. The following day, the brains were transferred to a 30% sucrose solution for preservation. Brains were sliced into 30 μm thick sagittal sections using a freezing microtome. Approximately three to four matched brain slices were used for immunostaining. The sections were washed and permeabilized in 0.3% Triton X‐100 in 1X PBS (PBST) following the antigen retrieval at 85°C for 10 min using 1X Decloaker (Biocare Medical, RV1000). Subsequently, sections were blocked at room temperature into 5% donkey serum in PBST for 1 h, later incubated at 4°C overnight with the following primary antibodies: NeuN (1:1000 in rabbit, Abcam ab104225), IBA1(1:250 in goat, Abcam ab5076), AT8 (1:500 in mouse, Invitrogen MN1020), AT180 (1:500 in mouse, Invitrogen MN1040), glial fibrillary acidic protein (GFAP) (1:500 in rat, Invitrogen 13‐0300). The next day, the sections were washed with PBST and stained with specific secondary antibodies tagged with AlexaFluor in 5% donkey serum for 1 h at room temperature (1:1000 anti‐rabbit, A10040; 1:500 anti‐goat, A21202; 1:500 anti‐mouse, A31571; 1:500 anti‐rat, 6430‐31). After an additional wash, the sections were carefully mounted onto the charged slides, underwent counterstaining, and then cover‐slipped with prolonged Antifade with DAPI (4′,6‐diamidino‐2‐phenylindole). Images were captured using a fluorescence microscope at consistent exposure and gains settings. Images were analyzed using ImageJ (National Institutes of Health [NIH], version 1.5.3r). Microglia (IBA1 positive cells) count density was analyzed using Imaris (Oxford instruments 10.0. 1) by quantifying voxels representing IBA1 positive cells structure within the region of interest, and the collective area was calculated by multiplying voxel count by size. The cell density was measured as cells per square micrometer (cells/ μm^2). Morphological analysis of microglia was performed on a total of two to three sections from three to four mice per genotype with an equal number of males and females. Brain tissue section images were acquired using the Nikon AR1 Confocal microscope at maximum intensity z‐stack acquisition with a 40X objective. Obtained images were processed using FIJI alongside the AnalyzeSkeleton plugin, as described by Young & Morrison, 2018.^16^ Briefly, images were transformed into binary, and refinement steps were utilized to remove unwanted elements. Skeletonized images were then analyzed to assess microglia morphological features. Data from skeletonized images were transferred to Excel for further analysis, including trimming, sorting, and calculating branch length and number of endpoints per cell.

### 2.5 Brain extract preparation

Cortical and hippocampal tissues from the left hemispheres were homogenized using tissue protein extraction reagent (T‐PER, Thermo Fisher Scientific, 78510) supplemented with 1X Halt cocktail of protease and phosphatase inhibitor (Thermo Fisher Scientific, 78440). A bicinchoninic acid (BCA, Pierce) Kit was used to measure the total protein concentration. The resulting lysates were aliquoted and stored at −80°C in a freezer. The supernatant was used for Meso‐Scale Discovery Assays and Western blotting. Pellets were subjected to extract insoluble tau fractions using the method adapted from described previously.^17^ Briefly, pellets were resuspended completely in 70% of formic acid, incubated at 150 rpm for 1 h at room temperature, and then neutralized with 1:3 dilution of neutralization buffer, following which they were vortexed and stored at 80°C in a freezer.

### 2.6 Cytokine panel assay

Left hemibrain cortical samples were prepared as described above. The samples were analyzed with two replicates (per sample) using the MSD proinflammatory cytokine panel (K15048D, MesoScale Discovery, Gaithersburg, MD, USA), a highly sensitive multiplex enzyme‐linked immunosorbent assay (ELISA). This panel measures levels of ten proinflammatory cytokines: interferon‐gamma (IFN‐γ), interleukin (IL)‐1β, IL‐2, IL‐4, IL‐5, IL‐6, IL‐10, IL‐12p70, KC/GRO, and tumor necrosis factor‐alpha (TNF‐α).

### 2.7 Phospho (T231) and Total tau measurement

Left hemibrain cortical and hippocampal soluble and insoluble samples were prepared as described above. The samples were analyzed with two replicates (per sample) using the MSD, Phospho (T231)/Total tau Kit (K152D, MesoScale Discovery, Gaithersburg, MD, USA), a highly sensitive ELISA. This assay explicitly measures Phospho (T231) and Total tau levels in the mouse brain homogenates.

### 2.8 Western blotting

The samples from left hemibrain cortical tissues were prepared as described above. Protein denaturation was carried out by heating samples at 100°C for 10 min in 4X NuPAGE LDS buffer (NP0007). Ten to forty micrograms of protein per sample were loaded onto 4%–12% NuPAGE Novex gels (Invitrogen) and electrophoresed at 150 V for 60 min. Proteins were transferred onto the PVDF membrane at 400 mA and blocked in 5% bovine serum albumin (BSA) prepared in Tris buffered saline (TBS) with 0.01% tween (TBST). The membranes were incubated with specific primary antibodies overnight at 4°C with gentle agitation. Western blotting was performed with the following primary antibodies: INPP5D (1:500 in rabbit, CST 2728), GAPDH (1:10,000 in rabbit, Invitrogen G8795), Tau5 (1:1000 in mouse, Abcam 80579), AT8 (1:1000 in mouse, Invitrogen MN1020), AT100 (1:1000, Invitrogen MN1060), PHF1(S396, S404) (1:10,000 in mouse, Peter Davies). After incubation with primary antibodies, membranes were washed with TBST and subsequently incubated with appropriate horseradish peroxidase (HRP) conjugated secondary antibodies diluted in 5% milk in TBST at room temperature for an hour, washed with TBST, developed in the ECL solution and imaged. Western blots images were analyzed using ImageJ.

### 2.9 Nanostring nCounter

Hippocampal tissues from the brain's left hemispheres were homogenized as described above. Equal volumes of homogenized tissue and RNA STAT‐60 (Tel‐Test Inc., CS‐502) were mixed, snap‐frozen, and preserved at −80°C until further use. RNA extraction was performed using the PureLink RNA mini kit (Invitrogen, 12183020) and the PureLink DNase (Invitrogen, 12185010) following the manufacturer's protocol. RNA quality and quantity were determined using the Nanodrop 2000 spectrophotometer (Thermo Fisher Scientific). Subsequently, according to the manufacturer's instructions, 200 nanograms of RNA from hippocampal tissue from 9‐month‐old mice were utilized for gene expression profiling with the nCounter Glia Profiling Panel (NANOSTRING TECHNOLOGIES). Differential gene expression analysis was done using the ROSALIND platform for nCounter analysis (ROSALIND Inc., version 3.35.5.0).[Fig alz14078-fig-0001]

### 2.10 RNA extraction and quantitative real‐time polymerase chain reaction

Left hemisphere cortical tissues were homogenized as described above. Equal volumes of homogenized tissue and RNA STAT‐60 (Tel‐Test Inc., CS‐502) were mixed, snap‐frozen, and preserved at −80°C until further use. RNA extraction was performed using the PureLink RNA mini kit (Invitrogen, 12183020) and the PureLink DNase (Invitrogen, 12185010) following the manufacturer's protocol. RNA quality and quantity were determined using the Nanodrop 2000 spectrophotometer (Thermo Fisher Scientific). A thousand nanograms of RNA were converted to cDNA using a high‐capacity RNA‐to‐cDNA kit (Applied Biosystems, 4399950). Following cDNA synthesis, a quantitative polymerase chain reaction (qPCR) was performed on a StepOne Plus Real‐Time PCR system (Life Technologies). Gene expression levels were assessed relative to *Gapdh* (Mm99999915_m1, Life Technologies) using the 2^−ΔΔCT^ method. Expressions of genes were quantified using the TaqMan Gene Expression Assay from Life Technologies (*Inpp5d* primer: Mm00494987; *Clec7a* primer: Mm01183349_m1; *Spp1* primer: Mm00436767_m1; *Mertk* primer: Mm00434920; *Axl* primer: Mm00437221_m1; *Lpl* primer: Mm00434764, *Pdcd1* primer: Mm01285676_m1, *Klrg1* primer: Mm00516879_m1, *Cd4* primer: Mm00442754_m1, *Cd8a* primer: Mm01182107_g1, *Cd3e* primer: Mm01179194_m1, *Cd3d* primer: Mm00442746_m1).[Fig alz14078-fig-0002]

### 2.11 Statistical analysis

Statistical analysis was performed using GraphPad Prism (Prism 10.0.3). Data presented as mean ± SEM. A Student's *t*‐test and non‐parametric one‐way analysis of variance (ANOVA) were performed with Tukey's post hoc test. A Pearson correlation coefficient was used for correlation analysis. The value of *p* < 0.05 was considered significant.

## 3 RESULTS

### 3.1 INPP5D protein expression is upregulated and positively correlated with pTau in the PS19 mouse model

**FIGURE 1 alz14078-fig-0001:**
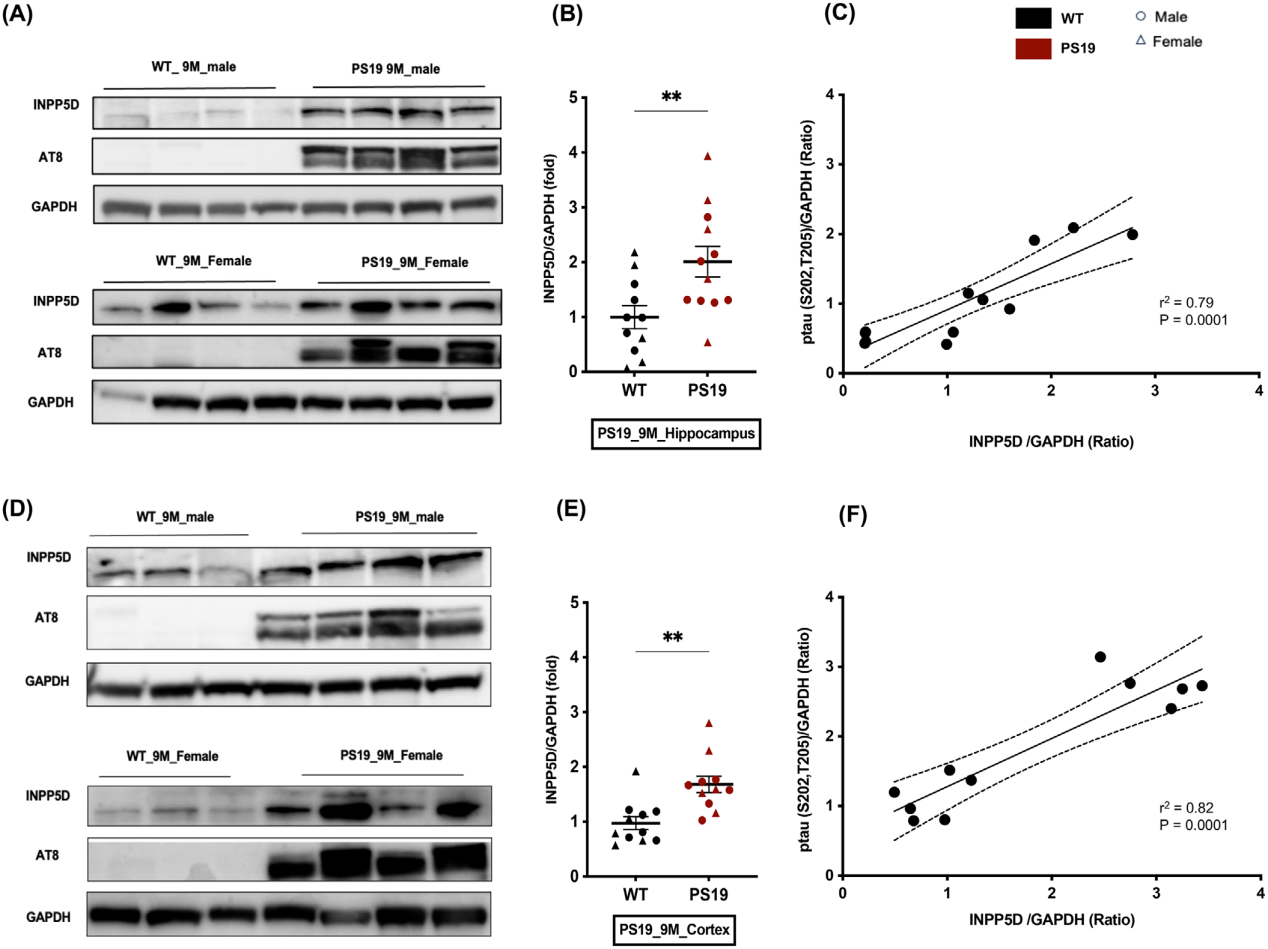
INPP5D protein expression is upregulated in PS19 mouse model of tau pathology. (A) Immunoblotting showing INPP5D and p‐tau AT8 expression in the hippocampus of 9‐month‐old wild‐type (WT) and PS19 mice. (B) Quantification of INPP5D expression. Expression of INPP5D was normalized with GAPDH. *N* = 11 (6 male, round symbol; 5 female, triangle symbol). (C) The expression level of INPP5D shows a positive correlation with p‐tau AT8 in the hippocampus of 9‐month‐old PS19 mice. (D) Immunoblotting showing INPP5D and p‐tau AT8 expression in cortices of 9‐month‐old WT and PS19 mice. (E) Quantification of INPP5D expression. Expression of INPP5D was normalized with GAPDH. *N* = 11 (6 male, round symbol; 5 female, triangle symbol). The student's *t*‐test was performed for statistical analysis. Data are represented as mean ± SEM (*p < 0.05 and ***p < 0.001). (F) The expression level of INPP5D shows a positive correlation with p‐tau AT8 in the cortex of 9‐month‐old PS19 mice. Pearson's correlation coefficient was performed to analyze the correlation between the expression level of INPP5D and p‐tau AT8. Expression of INPP5D and p‐tau AT8 was normalized to GAPDH. Each dot represents an individual sample.

Previous studies have demonstrated elevated expression of INPP5D in both AD human brains and the amyloid pathology mouse model, 5XFAD, with a positive correlation between INPP5D expression and amyloid burden.^6^, ^10^ To extend this understanding in the context of tau pathology, we conducted Western blot analysis for INPP5D expression in the PS19 mouse model of tau pathology,^15^, ^17^, ^18^, ^19^ which develops extensive tau pathology and motor deficits around 9 months of age.^15^ We examined the protein expression of INPP5D in the cortex and hippocampus at 9 months through blot, which was elevated in both regions compared to wild‐type (WT) mice (Figure 1A, B, D & E, Figure S1 A, C, E, & G). We also examined the expression of phosphorylated tau AT8 (S202/T205) on the same blot. We observed a positive correlation between the expression of INPP5D and AT8 in mice (Figure 1C, F, Figure S1 B, D, F, & H). Together, these results suggest that, like AD human brain data, elevated expression of INPP5D can be recapitulated in the PS19 mouse model and correlated positively with tau pathology. Therefore, the PS19 tau pathology mouse model was used to study the role of *Inpp5d* in tau pathology.[Fig alz14078-fig-0003]

### 3.2 *Inpp5d* haplodeficiency recovers motor deficits observed in PS19 mice

**FIGURE 2 alz14078-fig-0002:**
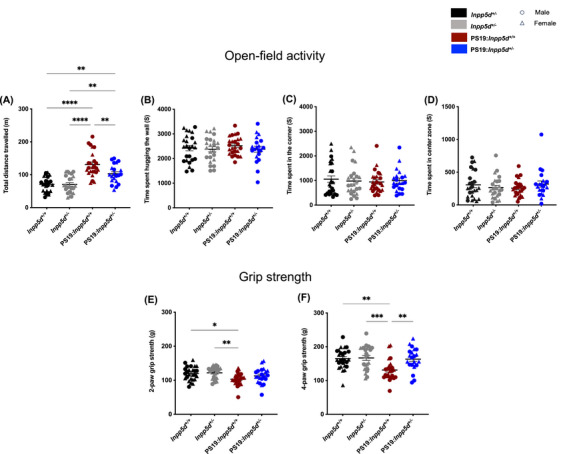
*Inpp5d* haplodeficienc*y* mitigates motor deficit observed in PS19 mice. (A) An open‐field test was performed to assess hyperactivity by calculating the total distance traveled by 9‐month‐old mice in the open‐field arena (*n* = 21–25 per genotype; male, round symbol; female, triangle symbol). The graph shows the total distance traveled by mice. (B, C, D) Anxiety‐like behavior was assessed by measuring time spent hugging the wall, time spent in the corner, and time spent in the center zone by mice in the open field arena. Graphs show time spent in the corner, time spent hugging the wall, and time spent in the center zone by mice. (E, F) A grip strength test was performed to assess the muscle strength of forelimbs (two‐paw) as well as combined forelimbs and hindlimbs (four‐paw) of 9 month‐old mice by using a grip‐strength meter (*n* = 21–25 per genotype, male, round symbol; female, Triangle symbol). A statistical test was performed using a one‐way analysis of variance (ANOVA) for total distance traveled, time spent hugging the wall, time spent in the corner, and two‐paw and four‐paw grip strength, followed by Tukey's post hoc test. Data are presented as the mean ± SEM (*p < 0.01, ***p < 0.001, and ****p < 0.0001).

The tau pathology mouse model, PS19, exhibits extensive tau pathology and motor deficits at 9 months.^15^, ^17^, ^18^, ^19^ We conducted an open‐field activity test that assessed hyperactivity and a grip strength test that assessed muscle strength in the mice.^15^ We compared haplodeficient *Inpp5d*:PS19 mice (PS19:*Inpp5d* ^+/−^) to WT *Inpp5d*:PS19 mice (PS19:*Inpp5d* ^+/+^), WT (*Inpp5d* ^+/+^), and haplodeficient *Inpp5d* mice (*Inpp5d* ^+/−^) to examine whether reduced expression of *Inpp5d* in PS19 mice modulates these motor deficits. We observed that PS19: *Inpp5d* ^+/−^ exhibited substantially reduced total distance traveled compared to PS19:*Inpp5d* ^+/+^mice (Figure 2A, Figure S2 A, E). No difference in total distance traveled was observed between *Inpp5d* ^+/+^ (WT) and *Inpp5d* ^+/‐^, suggesting that reducing *Inpp5d* expression in these mice showed no functional deficits at the baseline (Figure 2A). Furthermore, none of the genotypes displayed anxiety‐like behaviors, which were analyzed by measuring total time spent in the corner, hugging the wall (thigmotaxis) and time spent in the center zone, suggesting that reduction in *Inpp5d* expression did not induce anxiety‐like behaviors (Figure 2B, C, D, Figure S2 B‐H). The PS19 mice at 9 months of age show reduced muscle strength.^15^ Therefore, we measured grip strength for the two‐paws (front limbs) and four‐paws (four limbs) of the mice (Figure 2E, F, Figure S2 I—L) using the Grip strength meter. Significant improvement in four‐paw grip strength in haplodeficient‐*Inpp5d* PS19 mice was observed compared to PS19 mice (Figure 2E). No difference in the two‐paw (front limbs) and four‐paw (four limbs) of WT and *Inpp5d* ^+/‐^ mice was observed, similarly suggesting that reducing expression of *Inpp5d* showed no functional deficits at the baseline. Furthermore, since *Inpp5d* is a crucial regulator of immune response and our study utilized globally haplodeficient *Inpp5d* mice,^20^ we examined spleen weight in all genotypes of the mice at 9 months of age. We observed that spleen weight remains unchanged in *Inpp5d^+/‐^* and PS19:*Inpp5d* ^+/−^) mice (Figure S2 M—O), suggesting that haplodeficiency of *Inpp5d* may not exacerbate peripheral immune response at baseline as well as in PS19 mice. These results suggest that *Inpp5d* haplodeficiency in PS19 mice improved locomotor behavior rather than rescuing it and restored the four‐limbs grip strength at 9 months of age.

### 3.3 *Inpp5d* haplodeficiency exhibits no significant impact on the abundance of cellular occupancy but altered microglia morphology

**FIGURE 3 alz14078-fig-0003:**
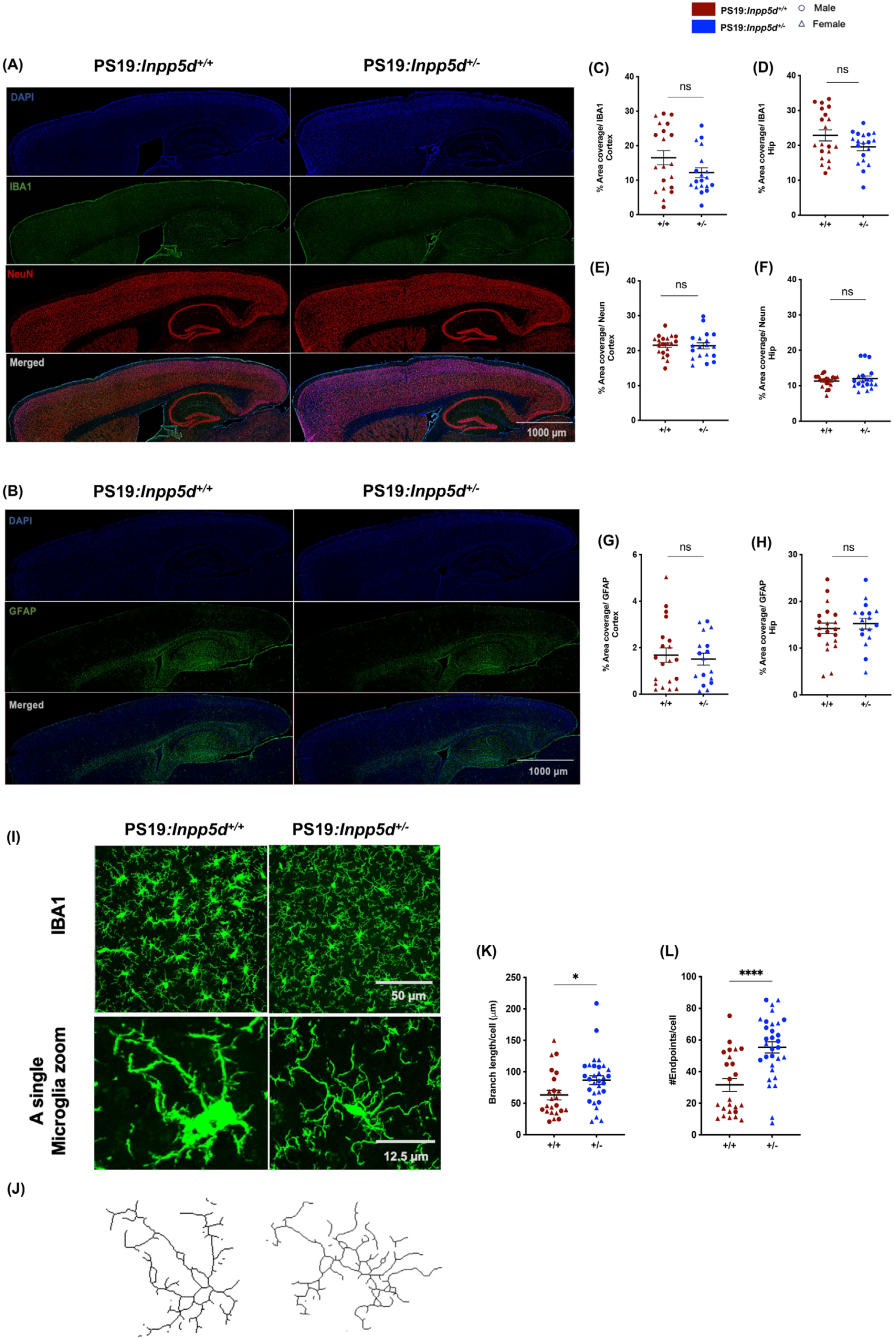
*Inpp5d* haplodeficiency exhibits no significant impact on the abundance of cellular occupancy but altered microglia morphology. (A, B) Immunostaining of microglia (IBA1, green), neuron (Neun, red), nuclei (DAPI, blue), astrocytes (GFAP, green) in the brain of 9‐month‐old PS19.*Inpp5d^+/+^* and PS19.*Inpp5d^+/−^* mice. Scale bar, 1000 μm. (C, D) Percentage area coverage of microglia (IBA1, green) determined in cortex and hippocampus. (E, F) Percentage area coverage of neurons (Neun, red) determined in cortex and hippocampus. (G, H) Percentage area coverage of astrocytes (GFAP, green) determined in cortex and hippocampus (*n* = 17–20 per genotype; male; round symbol; female; triangle symbol). (I) Representative immunohistochemical confocal images showing morphological characteristics of microglia extracted from the cortex of 9‐month‐old PS19.*Inpp5d^+/+^* and PS19.*Inpp5d^+/−^* mice and corresponding zoomed‐in view of a single microglia generated via ImageJ software. Scale bar, 50 μm. (J) Representation of skeletonized reconstructions of microglia morphological characteristics. (K) Quantification of branch length per cell, and (L) quantification of the number of endpoints per cell (*n* = 3–4 per genotype/male/female; male; round symbol, female; triangle symbol. Two to three sections per mouse were imaged; collectively 652 (PS19.Inpp5d+/+) and 668 (PS19.*Inpp5d^+/−^*) microglia were analyzed (student's *t*‐test was performed for statistical analysis for a percentage area coverage of IBA1, Neun, GFAP, quantification branch‐length, and number of endpoints/cell). Data are presented as the mean ± SEM.

Next, we quantified cell coverage of microglia, astrocytes, and neurons to gain insights into the broader cellular dynamic under the influence of reduced gene expression of *Inpp5d* in PS19 mice. We observed no change in staining coverage of IBA1 (microglia), NeuN (neurons) (Figure 3A, C, D, E & F, Figure S3 A‐H), and GFAP (astrocytes) (Figure 3B, G, H, Figure S3 I‐L). Furthermore, Since *Inpp5d* expression is restricted to microglia in the brain,^10^ we assessed microglia count density in the cortex and the hippocampus of 9‐month‐old *Inpp5d* ^+/+^, *Inpp5d* ^+/−^, PS19:*Inpp5d* ^+/+^, and PS19:*Inpp5d* ^+/−^ mice (Figure S3 M‐S). We observed no significant change in microglia density in the cortex of these mice, but we observed an increase in microglia density in the hippocampus of PS19:*Inpp5d* ^+/+^; however, haplodeficiency of *Inpp5d* in PS19 mice did not show a significant change in microglia density in the hippocampus. While cell coverage and density examination revealed no significant change with *Inpp5d* haplodeficiency, we further examined microglia morphological features. Surprisingly, we observed an increase in branch length and endpoints per microglia in PS19:*Inpp5d* ^+/−^ mice compared to PS19:*Inpp5d* ^+/+^ mice suggesting the complexity and function of microglia in response to haplodeficiency of *Inpp5d* (Figure 3I, J, K, L, Figure S4 A‐D). This suggests that the changes in the tau pathology and behavior outcomes might be independent of alteration in cell abundance or related to functional changes at the cellular level. However, further research is required to uncover precise changes.

### 3.4 *Inpp5d* haplodeficiency reduced tau pathology in PS19 mice

**FIGURE 4:**
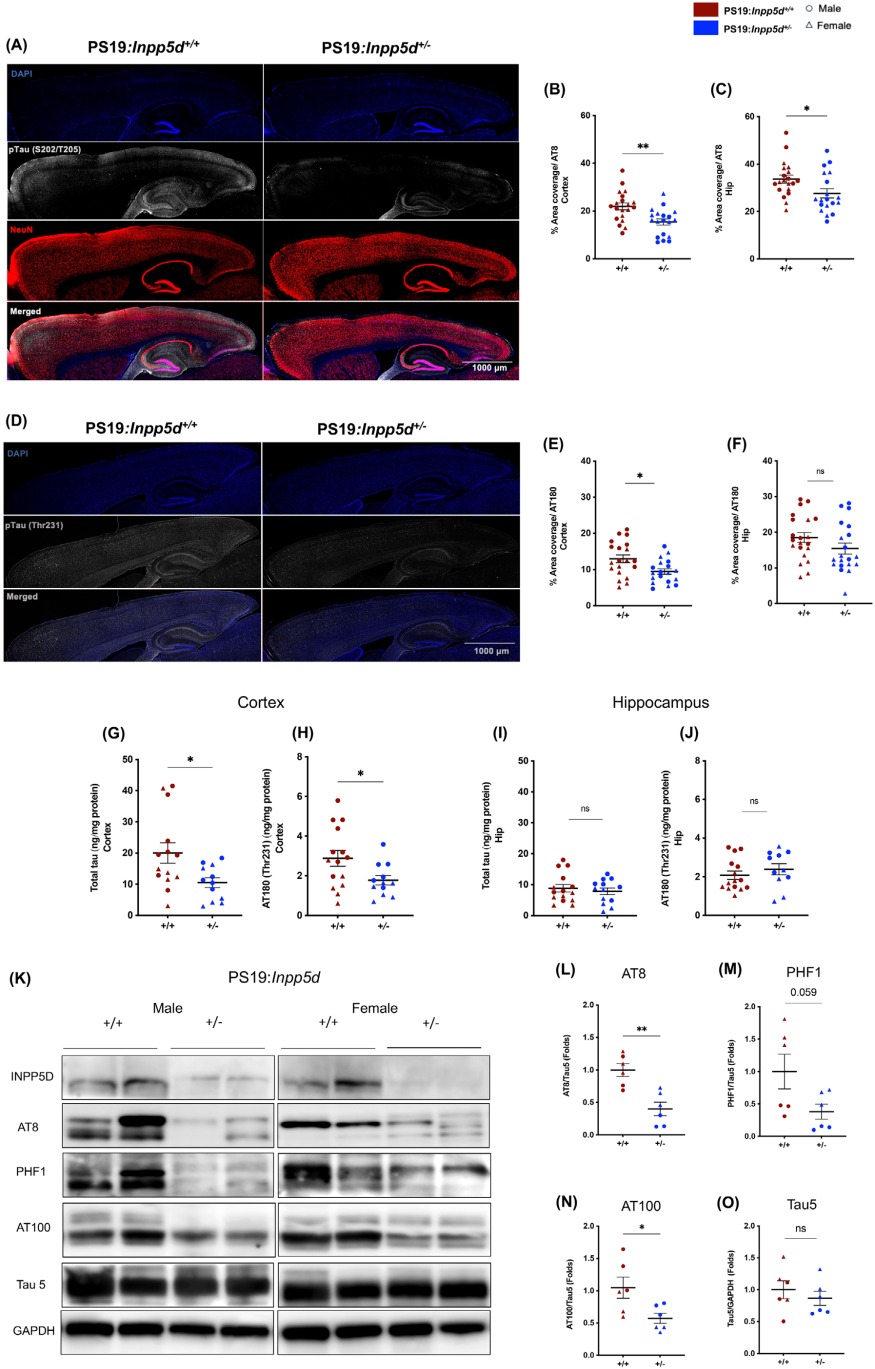
*Inpp5d* haplodeficienc*y* modulates tau pathology in PS19 mice. (A, D) Immunostaining of p‐tau AT8 (gray, A) and AT180 (gray, D), nuclei (DAPI, blue), neurons (NeuN, red) in the brain of 9‐month‐old PS19.*Inpp5d^+/+^* and PS19.*Inpp5d^+/‐^ mice*. Scale bar, 1000 μm. (B, C) Percentage area coverage of p‐tau AT8 was determined in cortex and hippocampus, respectively (*n* = 19–20 per genotype; male, round symbol; female, triangle symbol). (E, F) Percentage area coverage of p‐tau AT180 was determined in cortex and hippocampus, respectively (*n* = 19–20 per genotype; male, round symbol; female, triangle symbol). Statistical analysis was performed using the student's t‐test for a percentage area coverage of p‐tau AT8 and AT180. (G‐J) Total tau and p‐tau AT180 in cortices and hippocampus of 9‐month‐old PS19.*Inpp5d^+/+^* and PS19.*Inpp5d^+/‐^ mice* were measured by MSD (MesoScale Discovery) enzyme‐linked immunosorbent assay (ELISA) assay (*n* = 12–14 per genotype; male, round symbol; female, triangle symbol). (K) Immunoblotting shows expression of INPP5D, p‐tau AT8, PHF1, and tau 5 in the cortices of 9‐month‐old PS19.*Inpp5d^+/+^* and PS19.*Inpp5d^+/‐^* mice. (L‐O) Quantifications of p‐tau AT8, PHF1, and AT100 normalized with Tau5. Tau5 expression normalized to the GAPDH expression (*n* = 6 per genotype; male, round symbol; female, triangle symbol). A students' *t*‐test was performed for statistical analysis. Data are presented as the mean ± SEM (*p < 0.01, ***p < 0.001).

The PS19 mouse model demonstrates substantial tau phosphorylation and aggregation in various brain regions, including the hippocampus and cortex.^15^, ^17^, ^18^, ^19^ We aimed to examine whether *Inpp5d* haplodeficiency in PS19 mice influences tau pathology. To investigate this, we initially performed immunofluorescence staining of AT8 (S202/T205) and AT180 (Thr231) (Figure 4A, D) on the matched sagittal mouse brain sections. We observed substantially reduced staining of AT8 and AT180 in the cortex and hippocampus of PS19:*Inpp5d* ^+/‐^ mice compared to PS19 mice (Figure 4A, B, C, D, E & F, Figure S5 A‐H). However, for the AT180 staining, this alteration was only notable in the cortex and not the hippocampus in PS19: *Inpp5d^+/‐^* mice. We further performed the MesoScale Discovery ELISA assay to analyze total tau and phospho‐tau (Thr231) concentration in soluble and insoluble tau samples. Strikingly, Total tau and AT180 (Thr231) concentrations in the cortex were significantly reduced in PS19: *Inpp5d^+/‐^* mice (Figure 4G, H, Figure S5 I‐L). However, intriguingly, we observed an increase in AT180 (Thr231) concentrations in insoluble tau samples, indicative of tau aggregation (Figure S5 P). however, no significant difference was observed in AT180 (Thr231) concentrations between male and female groups considering insoluble fractions (Figure S5 Q, R). Despite this observation, a decrease in total tau and pTau levels in soluble fractions suggests a significant impact that may contribute to behavior and pathology in PS19:*Inpp5d* ^+/−^ mice. No change was observed in the concentration of total tau and AT180 in the hippocampus of the PS19:*Inpp5d^+/−^* mice (Figure 4I, J, Figure S5 S‐X). To further validate this analysis, we performed a Western blot for the phospho‐tau AT8 (S202/T205), AT100 (T212/S214), PHF1 (S396/S404), and total tau on the cortex lysates, and we observed a strong reduction in the expression of AT8, AT100, and PHF1 in PS19. *Inpp5d^+/‐^* mice (Figure 4K, L, M, N, & O, Figure S6 A‐H). These results suggest that *Inpp5d* haplodeficiency in PS19 mice influences tau pathogenesis and alleviates motor deficit in the PS19:*Inpp5d^+/‐^* mice.

### 3.5 *Inpp5d* haplodeficiency alters pro‐inflammatory cytokine levels

**FIGURE 5:**
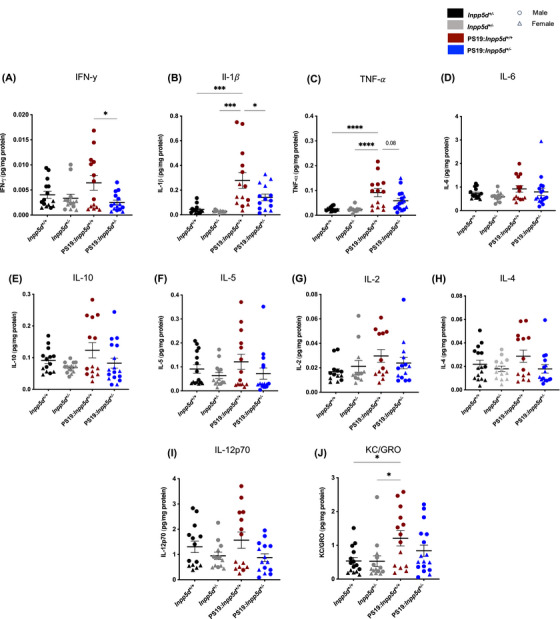
*Inpp5d* haplodeficienc*y* alters proinflammatory cytokine levels in PS19 mice. (A‐C) Graphs showing quantification levels of proinflammatory cytokines, interferon‐gamma (IFN‐γ), interleukin (IL) ‐1β, and tumor necrosis factor‐alpha (TNF‐α), measured using MSD assay in male mice. (D‐J). Graphs showing quantification levels of proinflammatory cytokines, IL‐6, IL‐10, IL‐5, IL‐2, IL‐4, IL‐12p70 & KC/GRO. Cytokine levels were normalized to total protein concentrations (*n* = 14–16 per genotype; male, round symbol; female, triangle symbol). A statistical test was performed using a one‐way analysis of variance (ANOVA), followed by Tukey's post hoc test. Data are presented as the mean ± SEM (*p < 0.01, ***p < 0.001, and ****p < 0.0001).

We further wanted to examine whether haplodeficiency of *Inpp5d* in PS19 mice influences proinflammatory cytokine levels. We performed a MesoScale Discovery multiplexed ELISA assay on the extract prepared from the cortical tissue of the mice brains. We detected a notable reduction of two pro‐inflammatory cytokine levels, IFN‐γ and IL‐1β (Figure 5A, B, Figure S7 A, B). We observed a trend of reduced‐level detection of TNF‐α (*p* = 0.084); however, it was insignificant (Figure 5C). We did not observe a significant difference in the level of seven other cytokines, that is, IL‐2, IL‐4, IL‐5, IL‐6, IL‐10, IL‐12p70, and KC/GRO (Figure 5D, E, F, G, H, I, & J, Figure S7 A, B). These data suggest that *Inpp5d* expression level may contribute to the modulation of proinflammatory cytokine levels in the presence of tau pathology in PS19 mice.

### 3.6 *Inpp5d* haplodeficiency led to gene expression alterations linked to immune response and cell migration

**FIGURE 6:**
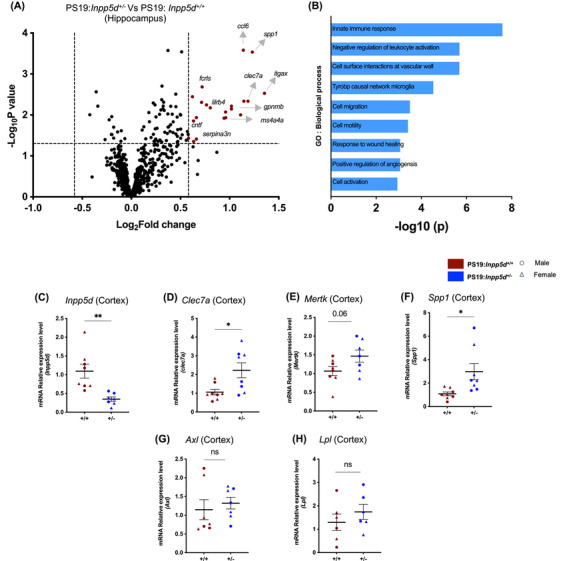
*Inpp5d* haplodeficienc*y* leads to gene expression alterations linked to immune response and cell migration. (A) Volcano plot showing differentially expressed genes (DEGs) (*p* < 0.05, FC > 1.5) in the hippocampus of 9‐month‐old PS19:*Inpp5d^+/‐^* versus PS19:*Inpp5d^+/+^ mice*. DEGs are analyzed using the nCounter Alzheimer's Disease and Glial Profiling panels from NanoString (*n* = 6 per genotype; male and female, increased expressed genes shown in red). (B) Identification of the top ontology terms of biological process among the upregulated genes within the hippocampus. (C) The graph shows reduced gene expression levels of *Inpp5d* in PS19.*Inpp5d^+/‐^* mice compared to PS19.*Inpp5d^+‐^* mice. (D‐F) The graphs show increased Clec7a, Mertk, and Spp1 gene expression levels in PS19.*Inpp5d^+/‐^* mice. (E, F) The graphs show unchanged gene expression levels for *Axl and Lpl* in PS19.*Inpp5d^+/‐^* mice. The student's *t*‐test was performed for statistical analysis. Data are presented as the mean ± SEM (*p < 0.01, ***p < 0.001).

Following the improvement in motor deficit outcomes and reduced tau pathology, we were next interested in assessing the comprehensive impact of *Inpp5d* haplodeficiency on gene expression patterns under tau pathology. A Nanostring Glia‐profiling panel was conducted on the hippocampal samples collected from six males and six females with genotypes PS19:*Inpp5d+/+* and PS19:*Inpp5d+/−* at 9 months of age. Differential gene expression analysis revealed upregulation of genes linked to cell migration and immune response in PS19:*Inpp5d+/‐* mice compared to PS19:*Inpp5d+/+* mice (Figure 6A). The analysis of gene ontology terms for biological processes on the differentially expressed genes (DEGs) showed modifications in various pathways (Figure 6B) and these enriched pathways in the hippocampus extended to processes associated with cell motility (*Itgax, Clec7a, Gpnmb, Ccl6, Spp1*), wound healing response (*Serping1, Cntf, Clec7a, Gpnmb*,), the Tyrobp causal network of microglia (*Itgax, Clec7a, Spp1, Cd84, Ms4a4a*) (Figure 6B). Following these observations, to further understand the gene expression changes associated with immune response in the cortex of PS19:*Inpp5d+/−* mice; we performed qPCR for specific genes, *Clec7a, Mertk, Spp1, Lpl*, and *Axl*, which have been implicated in the processes linked to immune response and cell migration and considered to have a significant impact in the progression of Alzheimer's disease^21^, ^22^, ^23^, ^24^, ^25^, ^26^ (Figure 6D, E, F, G, & H, Figure S8 A‐J). Despite unchanged gene expression of *Lpl* and *Axl*, we observed the upregulation of the genes *Clec7a, Spp1, and Mertk*, which also suggests an enhanced immune and cell migration‐related response in the cortex of PS19:*Inpp5d+/−* mice compared to PS19:*Inpp5d+/+* mice. We also measured *Inpp5d* gene expression, revealing a 50%–60% reduction in *Inpp5d* gene expression in PS19:*Inpp5d+/−* mice (Figure 6C). With recent findings indicating an interplay between T‐cell infiltration and microglia activation,^27^ coupled with our observation of increased immune response gene expression, we examined gene expression associated with T‐cell expressions such as *Pdcd1, Klrg1, Cd4, Cd8a*, and *Cd3d*. our results indicated no significant difference in gene expression levels associated with T‐cell expression (Figure S8 K, L, M, N, O & P). Unchanged expression of *Pdcd1, Klrg1, Cd4, Cd8a, Cd3d*, and *Cd3e* does not provide a direct association of T‐cell infiltration requiring further investigation for detailed understanding; however, it suggests that haplodeficiency of *Inpp5d* in PS19 mice may not contribute to influencing gene expression associated with T‐cell infiltration.

These results suggest that changes in the gene expression pattern due to *Inpp5d* haplodeficiency may contribute to modulating the microenvironment in the brain to promote a shift in tau‐related pathogenesis, potentially enhancing gene expression linked to cell migration and immune response. However, further research is required to understand the precise mechanisms underlying these gene expression changes.

## 4 DISCUSSION

In the present study, we investigated the role of *INPP5D*, an AD risk gene that exhibits specific expression in microglia within the brain, in the context of tau pathology. Previous studies have reported that INPP5D gene expression increases and correlates positively with amyloid pathology. Furthermore, recent publications have revealed that, in the amyloidogenic mouse model, inhibition of the *Inpp5d* gene ameliorates amyloid pathology and protects against neurodegeneration.^6^, ^10^, ^12^, ^14^ However, the role of *Inpp5d* in the context of tau pathology has been unexplored. Our study provides insights into how reducing the expression of *Inpp5d* under tau pathology leads to a notable improvement in several critical aspects of tau pathogenesis. We initiated this study due to our observation of a significant increase in INPP5D expression in the brains of tau pathology mice, PS19, emphasizing its significance through a positive correlation with ptau AT8 levels. This observation suggests that *Inpp5d* might be associated with modulating tau pathology.

Recently, our group and others have reported the impact of deficiency of *Inpp5d* in the mouse model of Alzheimer's disease that substantially exhibits amyloid pathology.^6^, ^12^, ^14^ These studies demonstrate that deficiency of *Inpp5d* facilitates microglia response toward Aβ plaques, mitigates amyloid pathology, and alleviates behavior impairments. In a parallel manner to what has been observed in these studies, our findings in the context of tau pathology demonstrate that *Inpp5d* haplodeficiency indicates a significant reduction in total tau accumulation and phosphorylation (Figure 4) as well as improved motor functions (Figure 2A, B) in the PS19 mouse model at 9 months of age. This study and a study by Lin et al.^6^ suggest that *Inpp5d* exerts a beneficial effect on the two primary hallmarks of AD pathology. Importantly, we did not observe any changes in the overall cell populations in PS19: *Inpp5d^+/−^* mice compared to PS19 *Inpp5d^+/+^* mice, suggesting that haplodeficiency of *Inpp5d* in PS19 mice may be tailored toward tau‐related pathogenesis or it may modulate cellular processes and microglia morphological features and complexity related to tau pathology without specifically affecting the overall cellular context. Elevated levels of proinflammatory cytokines such as IFN‐γ, IL‐1β, IL‐6, IL‐18, and TNF‐α have been shown to induce tau hyperphosphorylation and neuronal loss in AD and other neurodegenerative diseases.^24^, ^25^, ^26^ In PS19: *Inpp5d^+/−^* mice, we observed a reduction in IFN‐γ, IL‐1β, and TNF‐α cytokine levels (Figure 5), which are suggestive of a dampening neuroinflammatory response, potentially in response to reduced tau burden. Intriguingly, this reduction in the cytokine levels was pronounced in the male PS19: *Inpp5d^+/−^* mice compared to females. This sex‐specific difference may be attributed to the fact that female PS19: *Inpp5d^+/−^* mice do not exhibit an increase in the proinflammatory cytokine levels analyzed compared to *Inpp5d^+/+^* (WT) females. Thus, given the absence of robust proinflammatory phenotypes in PS19:*Inpp5d^+/+^* female mice, it is not surprising that *Inpp5d* haplodeficiency does not induce any observable effect. However, in experiments in which both female and male PS19:*Inpp5d^+/+^* mice exhibited significant pathological phenotypes, we observed that *Inpp5d* haplodeficiency leads to similar changes regardless of sex. These observations support the idea that the effects of *Inpp5d* haplodeficiency are only apparent in the presence of robust pathological phenotypes, which may be sex‐specific in PS19 mice, such as the level of inflammatory cytokines. In any case, it is notable that, when we combined male and female proinflammatory cytokine data, we observed a consistent trend of reduced proinflammatory cytokine levels in PS19: *Inpp5d^+/−^* mice. Altogether, this reduction also aligns with the overall improvement of motor deficits.

Interestingly, the enrichment analysis of gene ontology terms for biological processes on the DEGs revealed modifications in various immune responses and cell motility pathways in the hippocampus (Figure 6). Upregulation in genes such as *Clec7a, Itgax, serping1, Lgals3bp, Fclrls Gpnmb, Mertk, Lilrb4*, and *Ms4a4a*, which have been implicated as associated with microglia, emphasizes the targeted involvement of immune‐response‐related genes in PS19: *Inpp5d^+/−^* mice. A recent study^22^ in the context of tau accumulation has shown higher levels of certain genes such as *Mertk*, *Gas6*, *Lpl, Cst7*, and *Csf1* are associated with reduced tau accumulation and less cognitive decline in individuals affected by both amyloid beta and tau pathology. Furthermore, in a 12‐month tau‐seeded mouse model study,^23^ mice observed at 3 and 5 months post‐seeding revealed that upregulation of genes such as *Clec7a*, *Cts7*, and *Ctsd* correlates with reduced hyperphosphorylated tau levels. While our observation of gene expression patterns may not precisely match these studies,^22^, ^23^ they suggest potential alignment in gene expression patterns and tau pathology dynamics. In addition to the alterations in the immune response‐related genes, we also observed distinct profiles of DEGs intricately linked to cell migration, cell motility, angiogenesis, and wound healing. These alterations may reflect a concerted effort by microglia and different cell populations to respond to tau pathology in PS19: *Inpp5d^+/−^* mice. Further evaluation of these specific genes is essential to understand the mechanisms by which they collectively contribute to mitigating tau pathology.

Taken together, our data show the association of *Inpp5d* gene functions with tau pathology and suggests that *Inpp5d* haplodeficiency provides an avenue for further evaluation as a therapeutic potential for mitigating tau pathology and improving motor deficits.

## 5 CONCLUSION

Our findings demonstrated that *Inpp5d* haplodeficiency in PS19 mice attenuated tau pathology and improved motor functions. *Inpp5d* haplodeficiency is associated with modulating proinflammatory cytokine levels and immune‐response‐related gene expression patterns, highlighting the concerted microglia and different cellular responses to tau pathology. These findings provide insights into the complex interplay between *Inpp5d*, behavior, and tau pathology. However, they require further mechanistic investigations and provide the foundation for further evaluation of therapeutic interventions aimed at *INPP5D* inhibition, which may benefit tauopathies.

## AUTHOR CONTRIBUTIONS

Disha M. Soni and Adrian L. Oblak conceived and designed the study. Disha M. Soni conducted the experiments and analyzed the data. Peter Bor‐Chian Lin assisted with the WB and Nanostring_glia profiling. Christopher D. Lloyd assisted with the IHC experiment. Disha M. Soni, S.C., Bruce T. Lamb, and Adrian L. Oblak interpreted and discussed the results. Disha M. Soni and Adrian L. Oblak wrote the manuscript with critical revisions from Shaoyou Chu, Miguel Moutinho, Emily Mason, and Bruce T. Lamb. All authors reviewed and approved the manuscript.

## CONFLICT OF INTEREST STATEMENT

The authors declared no conflict of interest in this study. Author disclosures are available in the supporting information.

## CONSENT STATEMENT

No human subject materials were utilized in this study.

## Supporting information

Supporting Information

Supporting Information
