# Supplementary material for: Inpp5d haplodeficiency alleviates tau pathology in the PS19 mouse model of Tauopathy
Source: Alzheimers Dement. 2024 Jun 26;20(7):4985–98. doi: 10.1002/alz.14078 (PMC11247686; doi:10.1002/alz.14078)
Supplement: Supplementary file 1 — Supporting Information [file ALZ-20-4985-s001.pdf]

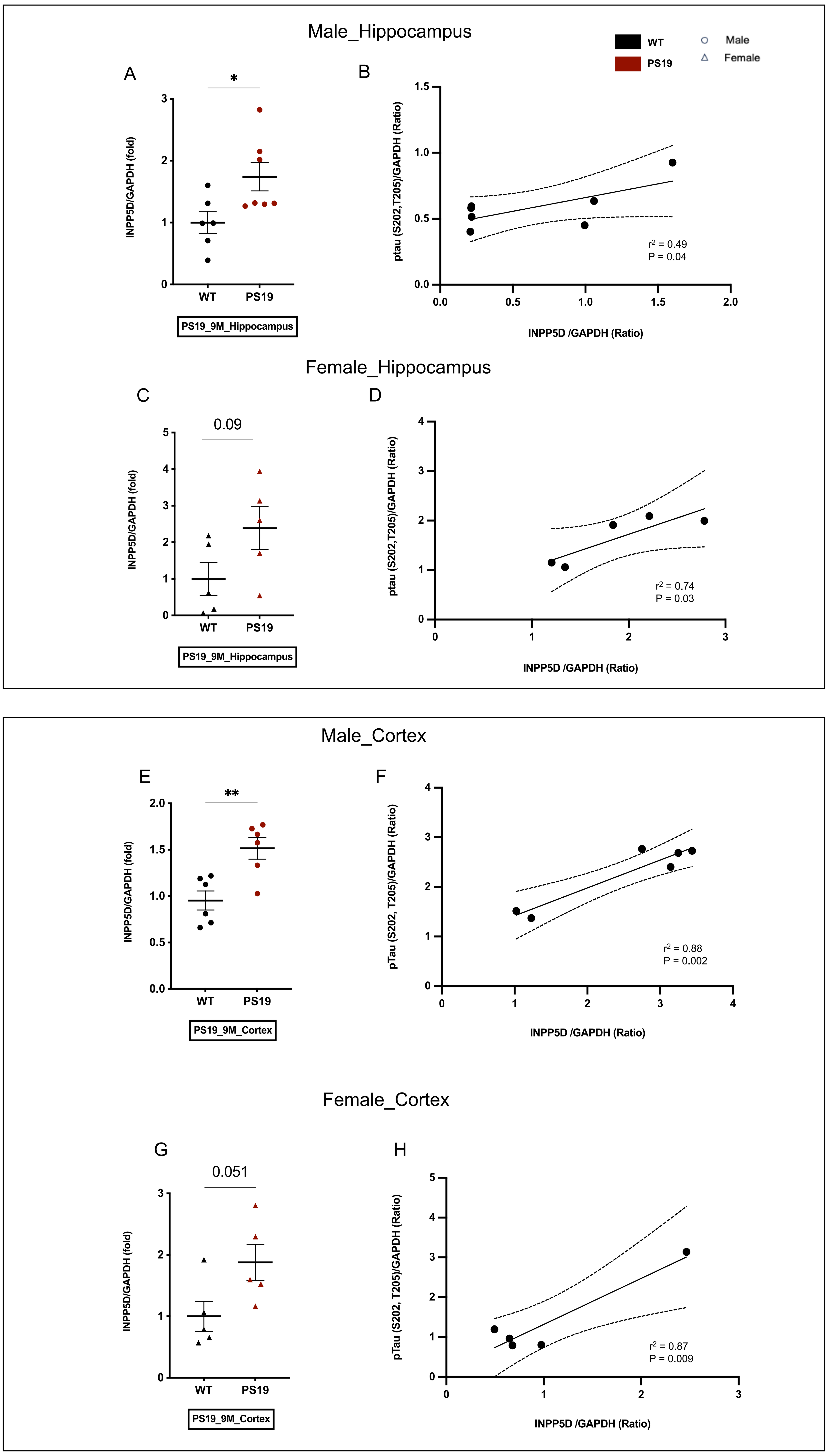

Supplementary Figure S1. INPP5D protein expression upregulated in PS19 mouse model of tau pathology.
(A) Quantification of INPP5D expression in PS19 male mice in the hippocampus. INPP5D expression was normalized with GAPDH. (B) The expression level of INPP5D shows a positive correlation with p-tau AT8 in the hippocampus of 9-month-old PS19 male mice. (C) Quantification of INPP5D expression in the hippocampus of PS19 female mice. Expression of INPP5D was normalized with GAPDH. N=11 (6 Male (Round symbol), 5 Female (Triangle symbol). (D) The expression level of INPP5D shows a positive correlation with p-tau AT8 in the hippocampus of 9-month-old PS19 female mice. (E) Quantification of INPP5D expression in PS19 male mice in the cortex. Expression of INPP5D was normalized with GAPDH. (F) The expression level of INPP5D shows a positive correlation with p-tau AT8 in the cortex of 9-month-old PS19 male mice. N=11 (6 Male (Round symbol), 5 Female (Triangle symbol) (G) Quantification of INPP5D expression in PS19 female mice in the cortex. Expression of INPP5D was normalized with GAPDH. (H) The expression level of INPP5D shows a positive correlation with p-tau AT8 in the cortex of 9-month-old PS19 female mice. Pearson's correlation coefficient was performed to analyze the correlation between the expression level of INPP5D and p-tau AT8. Expression of INPP5D and p-tau AT8 was normalized to GAPDH. Each dot represents an individual sample. The students' t-test was performed for statistical analysis. Data are represented as mean ± SEM (*$p < 0.05$ and ***$p < 0.001$).

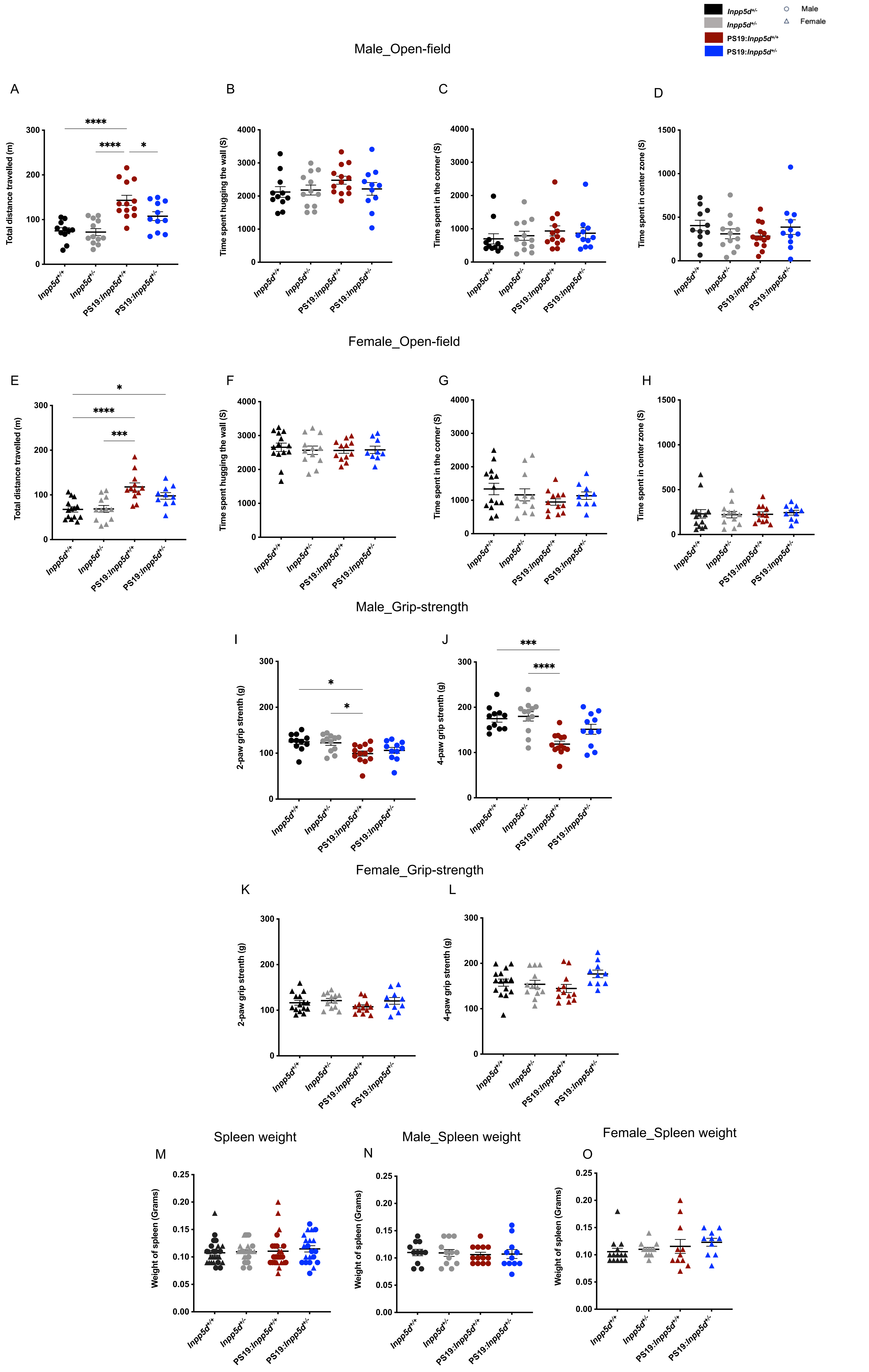

Supplementary Figure S2. *Inpp5d* haplodeficiency recovered motor deficit in PS19 mice.
(A,E) The open-field test assessed hyperactivity by calculating the total distance traveled, (B, F) Time spent hugging the wall, (C, G) Time spent in the corner (D, H) Time spent in the center zone by 9-month-old mice in the open-field arena (n=21-25 per genotype; male, round symbol; female, Triangle symbol. (I, K) A grip strength test was performed to assess the muscle strength of forelimbs (2-paw), (J, L) combined forelimbs and hindlimbs (4-paw) of 9 months-old mice by using a Grip-strength meter (n=21-25 per genotype, male, round symbol; female, Triangle symbol). (M) Graphs illustrate the measurement of Spleen weight obtained using a calibrated weighing machine in 9-month-old mice, (N) male, (O) Female (n=21-25 per genotype, male, round symbol; female, Triangle symbol presented separately as individual graphs). A statistical test was performed using a one-way analysis of variance (ANOVA) for the open field test and grip strength test, followed by Tukey's post hoc test. Data are presented as the mean ± SEM (*p< 0.01, ***p < 0.001 and ****p < 0.0001).

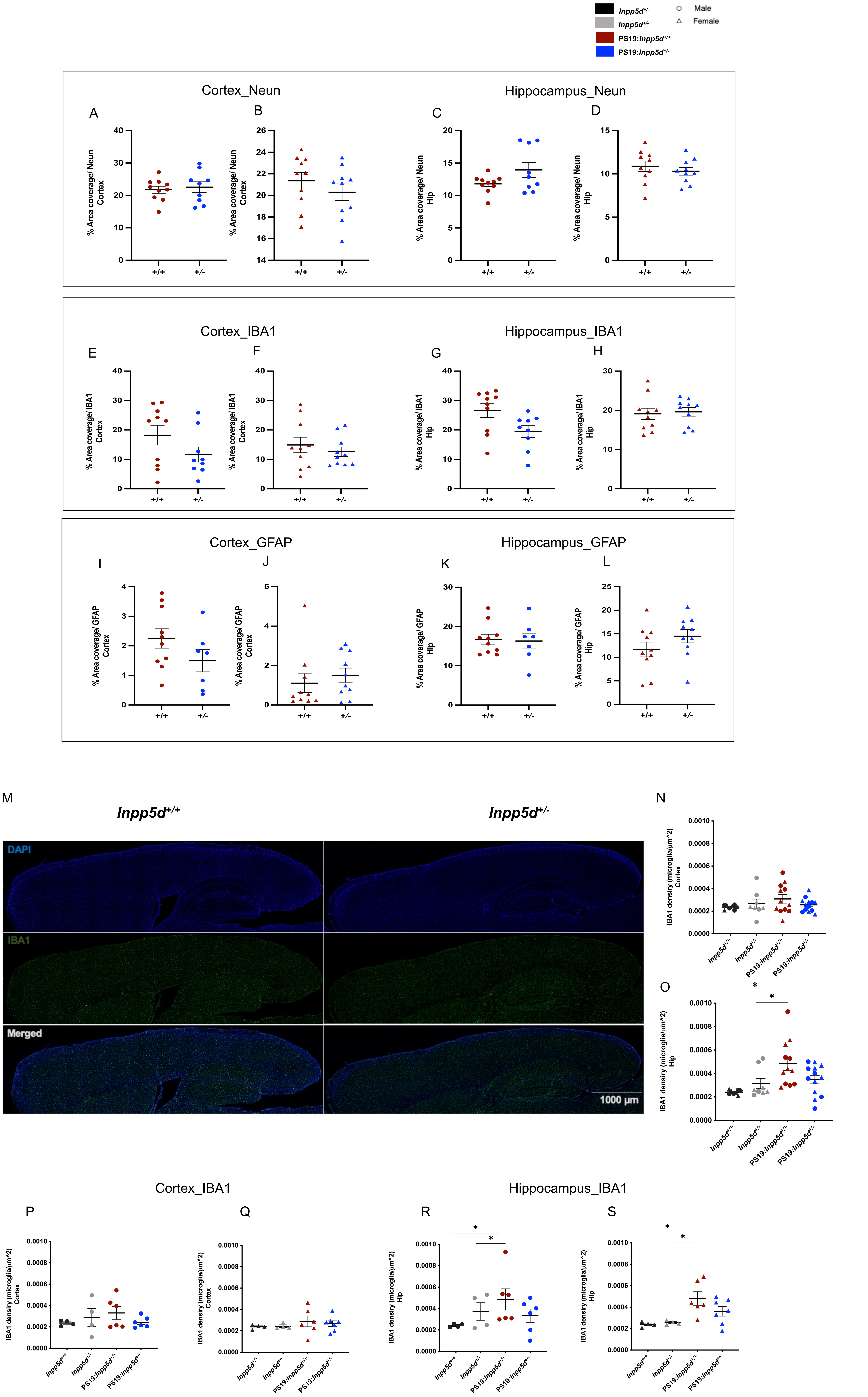

Supplementary Figure S3. *Inpp5d* haplodeficiency exhibits no significant impact on the coverage of cells in PS19 mice. (A, B, C, D) % Area coverage of neurons (Neun) determined in cortex and hippocampus. (E, F, G, H) % Area coverage of microglia (IBA1) determined in cortex and hippocampus. (I, J, K, L) % Area coverage of astrocytes (GFAP) determined in cortex and hippocampus (n=17-20 per genotype, male; round symbol, Female; triangle symbol presented separately as individual graphs, student's t-test was performed for statistical analysis). (M) Immunostaining of microglia (IBA1, green), and nuclei (DAPI, blue) in the brain of 9-month-old *Inpp5d*$^{+/+}$ and *Inpp5d*$^{+/-}$ mice. Scale bar, 1000 µm. (N, O) Quantification of density of microglia (IBA1, green) determined in the cortex and hippocampus. (n=8-13 per genotype, male; round symbol, Female; triangle symbol). (P, Q) Quantification of density of microglia (IBA1, green) determined in the cortex and (R, S) in the hippocampus. (male; round symbol, Female; triangle symbol presented separately as individual graphs). (A statistical test was performed using a one-way analysis of variance (ANOVA) for a cell count density). Data is presented as the mean ± SEM.

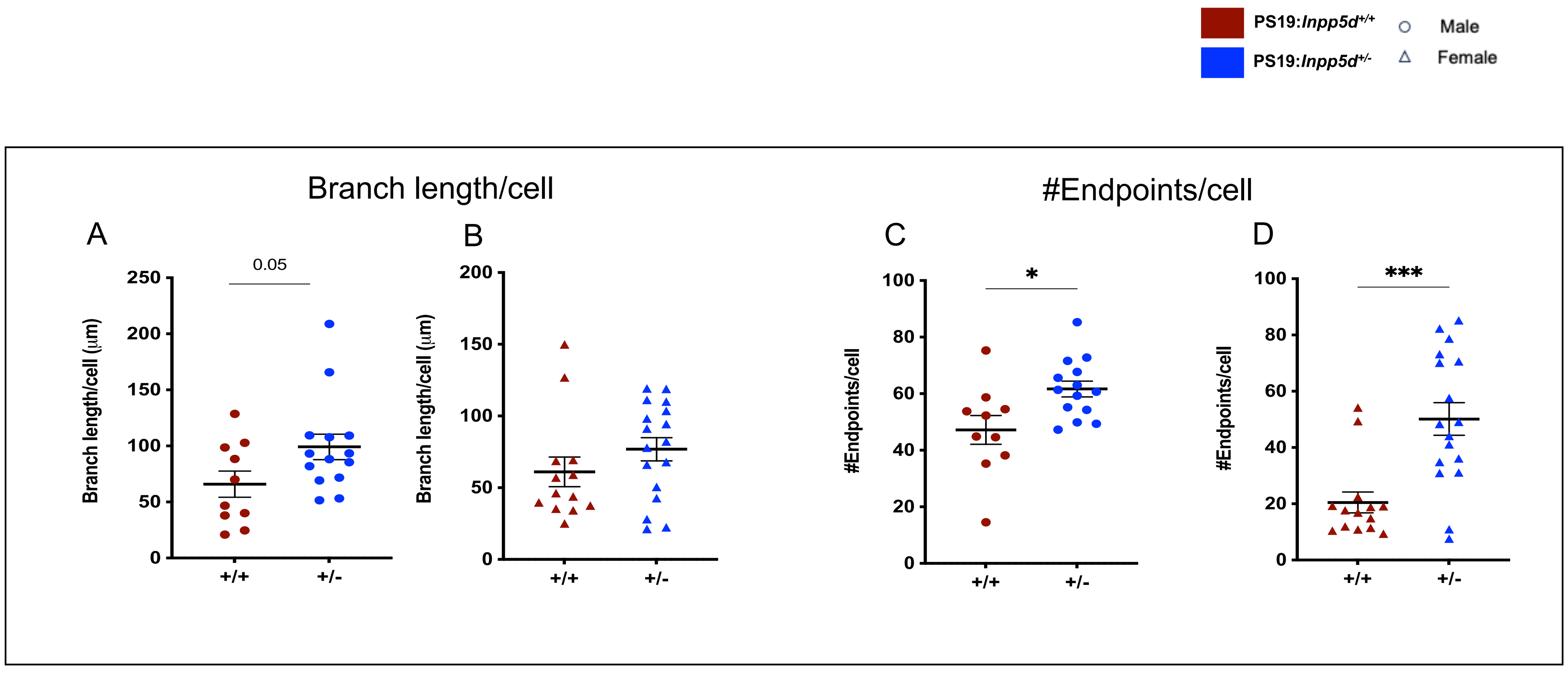

Supplementary Figure S4. ***Inpp5d*** **haplodeficiency impacts microglia morphology in PS19 mice.** (A, B) Quantification of branch length/cell, and (C. D) Quantification of endpoints/cell. (n=3-4 per genotype/male/female, male; round symbol, Female; triangle symbol presented separately as individual graphs) A student's t-test was performed for statistical analysis. A statistical test was performed using a one-way analysis of variance (ANOVA) for a cell count density. Data is presented as the mean ± SEM.

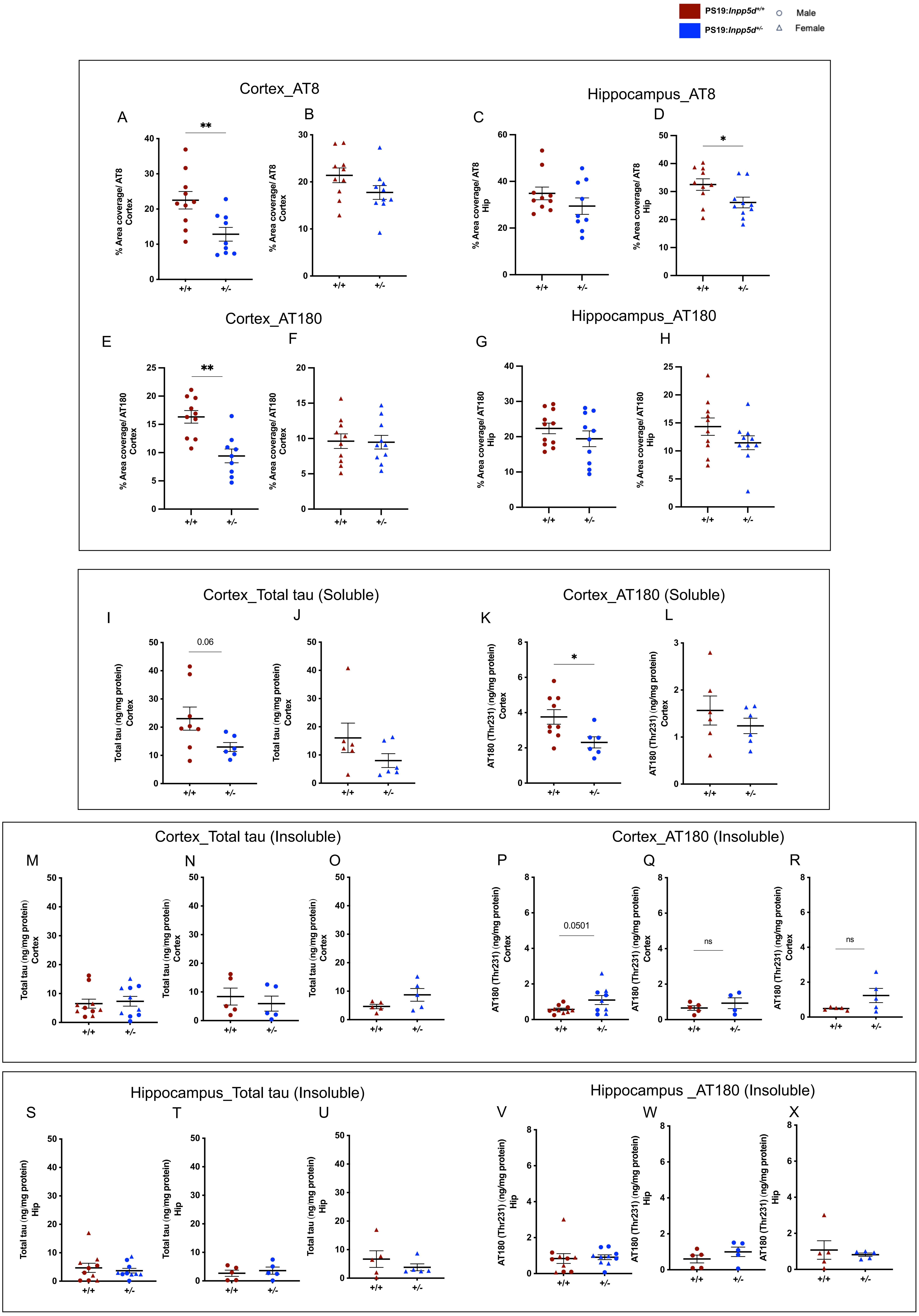

Supplementary Figure S5. *Inpp5d* haplodeficiency modulates tau pathology in PS19 mice.
(A, B, C, D) % Area coverage of p-tau AT8 was determined in cortex and Hippocampus, respectively (n=19-20 per genotype, male; round symbol, Female; triangle symbol). (E, F, G, H) % Area coverage of p-tau AT180 was determined in Hippocampus, respectively (n=19-20 per genotype, male; round symbol, Female; triangle symbol presented separately as individual graphs). (I, J, K, L) Quantification of total tau and p-tau AT180 in soluble fractions of cortices of 9 months-old PS19.*Inpp5d*$^{+/+}$ and PS19.*Inpp5d*$^{+/-}$ *mice* were measured by MSD ELISA assay. (n=12-14 per genotype, male; round symbol, Female; triangle symbol presented separately as individual graphs). (M) Quantification of total tau and (P) p-tau AT180 in Insoluble fractions of cortices of 9 months-old PS19.*Inpp5d*$^{+/+}$ and PS19.*Inpp5d*$^{+/-}$ *mice* (N, O) total tau and (Q, R) p-tau AT180 in insoluble fractions of male and female cortices presented separately as individual graphs. (n=9-12 per genotype, male; round symbol, Female; triangle symbol). (S) Quantification of total tau and (V) p-tau AT180 in Insoluble fractions of hippocampus of 9 months-old PS19.*Inpp5d*$^{+/+}$ and PS19.*Inpp5d*$^{+/-}$ *mice* (T, U) total tau and (W, X) p-tau AT180 in insoluble fractions of male and female hippocampus presented separately as individual graphs. (n=9-12 per genotype, male; round symbol, Female; triangle symbol). Statistical analysis was performed using the student’s t-test for a % area coverage of p-tau AT8 and AT180

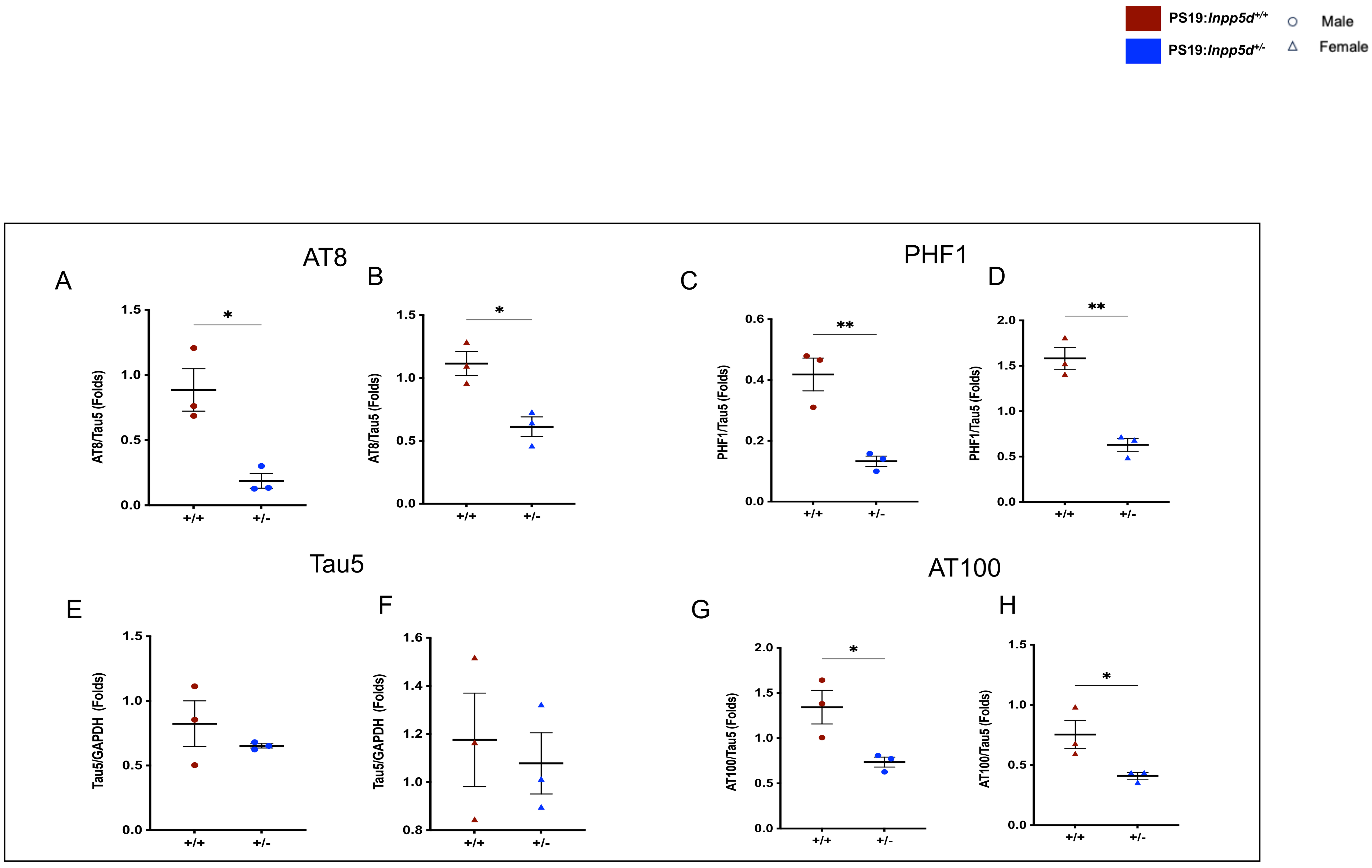

Supplementary Figure S6. *Inpp5d* haplodeficiency modulates pTau levels in PS19 mice.
(A, B) Quantifications of p-tau AT8, (C, D) PHF1, (E, F) Tau5 normalized to the expression of GAPDH, and (G, H) AT100 normalized to Tau5 (n=6 per genotype, male; round symbol, Female; triangle symbol presented separately as individual graphs). A students' t-test was performed for statistical analysis. Data is presented as the mean ± SEM (*p< 0.01, ***p < 0.001).

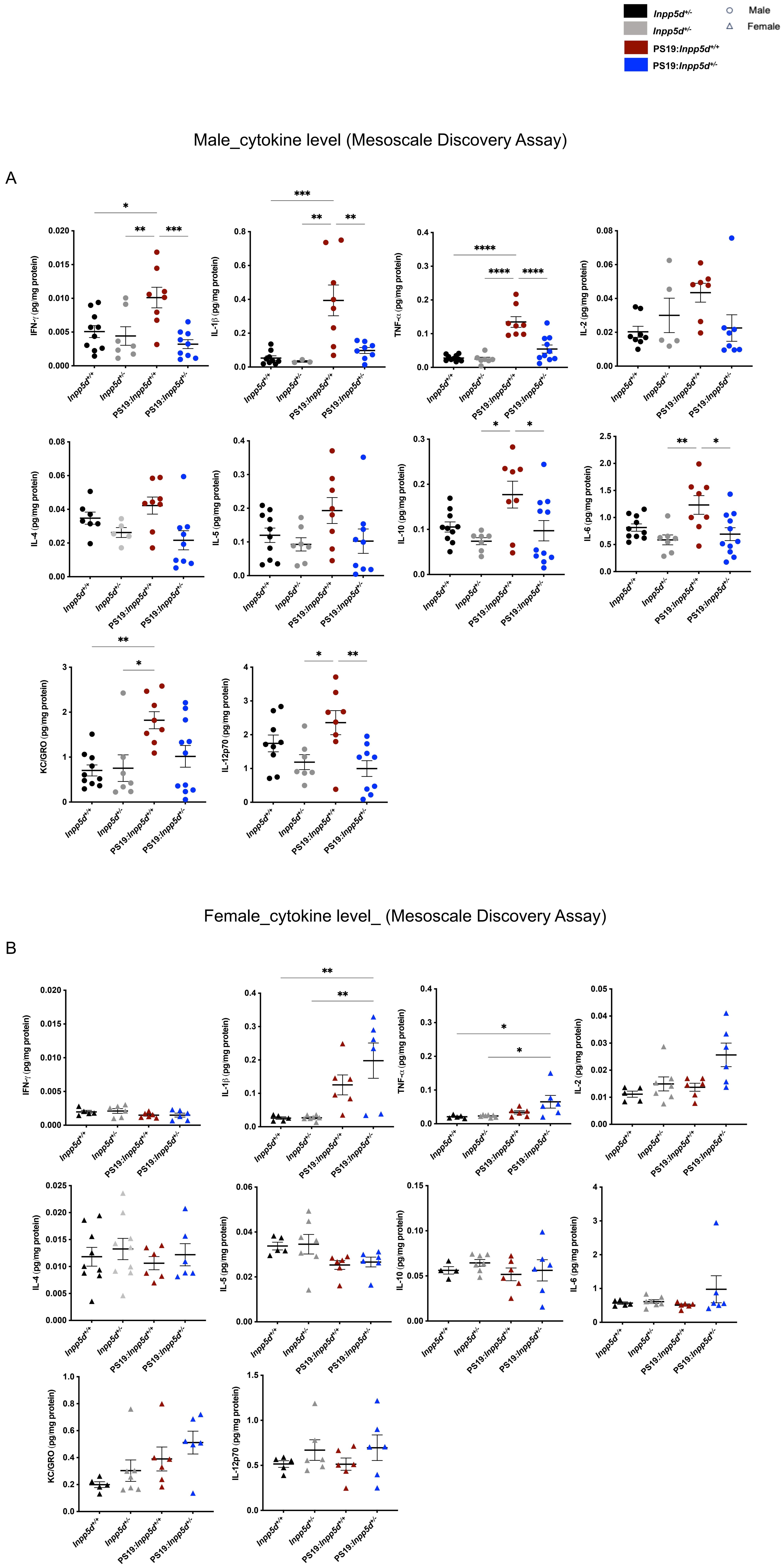

Supplementary Figure S7. *Inpp5d* haplodeficiency alters cytokine levels in PS19 mice.

(A) Quantifications of levels of proinflammatory cytokines measured using MSD assay in male mice. (B) Quantifications of levels of proinflammatory cytokines measured using MSD assay in females. The cytokine levels were normalized to total protein concentrations.). A statistical test was performed using a one-way analysis of variance (ANOVA), followed by Tukey's post hoc test. Data are presented as the mean ± SEM (*p< 0.01, ***p < 0.001 and ****p < 0.0001).

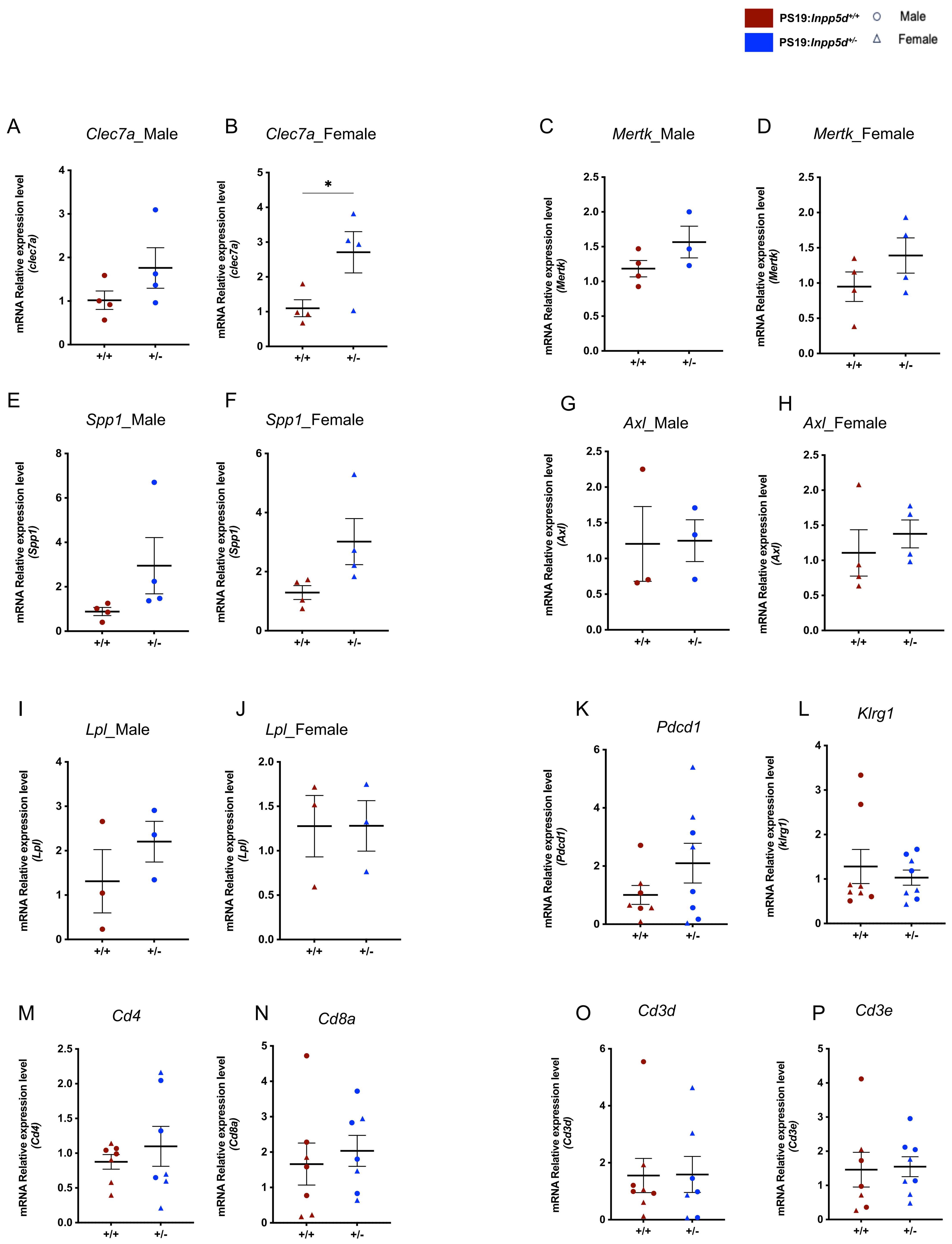

Supplementary Figure S8. *Inpp5d* haplodeficiency leads to gene alterations linked to immune response and cell migration.
(A, B) The graphs show an increase in mRNA expression of clec7a in PS19: *Inpp5d*$^{+/-}$ male and female mice, respectively. (C, D) The graphs show an increase in mRNA expression of *Mertk* in PS19: *Inpp5d*$^{+/-}$ male and female mice, respectively. (E, F) The graphs show increased mRNA expression of *Spp1* in PS19: *Inpp5d*$^{+/-}$ male and female mice, respectively. (G, H) The graphs show (I, J) *Axl* and *lpl* mRNA expression in PS19: *Inpp5d*$^{+/-}$ male and female mice, respectively. (K) The graphs show *Pdcd1,* (L) *Klrg1* (M) *Cd4* (N) *Cd8a* (O) *Cd3d* (P) *Cd3e* mRNA expression in PS19: *Inpp5d*$^{+/-}$ mice, respectively. (n=3,4 per genotype, male; round symbol, Female; triangle symbol). A students' t-test was performed for statistical analysis. Data are presented as the mean ± SEM (*p< 0.01, ***p < 0.001).
